# Transitions between classes of neuronal excitability and bifurcations induced by autapse

**DOI:** 10.1038/s41598-017-07051-9

**Published:** 2017-07-28

**Authors:** Zhiguo Zhao, Huaguang Gu

**Affiliations:** School of Aerospace Engineering and Applied Mechanics, Tongji University, Shanghai, 200092 China

## Abstract

Neuronal excitabilities behave as the basic and important dynamics related to the transitions between firing and resting states, and are characterized by distinct bifurcation types and spiking frequency responses. Switches between class I and II excitabilities induced by modulations outside the neuron (for example, modulation to M-type potassium current) have been one of the most concerning issues in both electrophysiology and nonlinear dynamics. In the present paper, we identified switches between 2 classes of excitability and firing frequency responses when an autapse, which widely exists in real nervous systems and plays important roles via self-feedback, is introduced into the Morris-Lecar (ML) model neuron. The transition from class I to class II excitability and from class II to class I spiking frequency responses were respectively induced by the inhibitory and excitatory autapse, which are characterized by changes of bifurcations, frequency responses, steady-state current-potential curves, and nullclines. Furthermore, we identified codimension-1 and -2 bifurcations and the characteristics of the current-potential curve that determine the transitions. Our results presented a comprehensive relationship between 2 classes of neuronal excitability/spiking characterized by different types of bifurcations, along with a novel possible function of autapse or self-feedback control on modulating neuronal excitability.

## Introduction

Neuronal electronic activities, such as firing and resting states, play basic and important roles in achieving biological functions of the nervous system^1, 2^ (for example, information encoding, transmission, and processing^2–5^). Early in 1948, Hodgkin distinguished different firing frequency responses of the resting state to external constant depolarization current stimulations, and then defined 3 classes of neuronal excitability^6^. As the depolarization current increases, the firing frequency continuously increases from 0 to a certain non-zero value for class I excitability, whereas it switches from 0 to a nearly fixed value for class II excitability. Except for the firing frequency response to external constant current, class I and II neurons exhibit different phase response curves to excitatory impulsive perturbations^7, 8^, different frequency response properties (integrator or resonator) to periodic stimulus^2, 9^, different coefficients of variation, or different histograms of interspike intervals to noise or stochastic stimulus^10–12^. Furthermore, neuronal excitabilities can affect spatiotemporal behaviors of the nervous system. For example, it is easier to obtain synchronization for a neuronal network with class II neurons than that with class I neurons^7, 13–15^. Neurons with class I and II excitabilities have been observed in biological experiments. For example, pyramid neurons in the hippocampus exhibit class I excitability^2, 16^, while interneurons in the neocortical and entorhinal cortex manifest class II excitability^15, 17, 18^.

In nonlinear dynamics, a relationship between neuronal excitability and bifurcation was built^1–3^. For example, class I excitability corresponds to a resting state (stable equilibrium) changed to firing (limit cycle) via saddle-node on invariant circle (SNIC) bifurcation as the depolarization current increases; meanwhile, class II excitability corresponds to Andronov-Hopf bifurcations^1–3^, which have been widely investigated in the classical 2-dimensional Morris-Lecar (ML) model with variables (*V*, *w*)^3, 16, 19–21^. The position relationship between 2 nullclines is different for the 2 classes of excitability. The nullcline $\dot{V}=0$ is tangent to $\dot{w}=0$ at saddle-node for class I excitability, and intersects $\dot{w}=0$ at focus for class II excitability. Furthermore, the transitions between 2 classes of neuronal excitability can be well interpreted by the high-codimension bifurcations. For example, a codimension-2 Bogdanov-Takens bifurcation^1–3, 19–28^ related to both Hopf and saddle-node bifurcations, and a codimension-3 bifurcation^29, 30^ (degenerate pitchfork bifurcation) have been investigated. In addition, the classes of spiking^2^, which are characterized by firing frequency responses to a decrease in depolarization current, have attracted research attention. For example, if the firing or spiking is changed to resting state via an SNIC as the depolarization current is decreased, the spiking is named as class I since the spiking exhibits a nearly zero frequency. If the firing or spiking is changed to resting state via a fold bifurcation of limit cycle (LPC) near a subcritical Hopf (SubH) bifurcation point, the spiking is called class II because of a non-zero firing frequency. The classes of spiking corresponding to bifurcations of the limit cycle, combined with the classes of excitability, are helpful for understanding the dynamics of transitions between resting and firing states^1–3, 19–28^.

In biophysics, neuronal excitability is related to the balance between different ionic currents and the competition between inward and outward currents, especially for mammalian central neurons with rich ion channels, which is characterized by current-voltage (I–V) curves^16, 19, 28–30^. For SNIC, net current at perithreshold potentials is inward at steady-state (the steady-state I–V curve is non-monotonic), but for SubH, net current at steady state is outward (the steady-state I–V curve is monotonic decreases), and the inward current activates are faster than outward current activities^16, 19^. The I–V curves have relationships to the excitability classes^2, 16, 19^. For example, the increase in the outward current contributed by the M-type potassium current can well interpret the transition from class I excitability *in vitro* to class II excitability under *vivo*-like condition^16^. The increase of A-type potassium conductance or the decrease of calcium conductance, which corresponds to the increase in the outward current or decrease in the inward current, respectively, can both induce the transition from class II to class I, and vice versa^31^.

The modulations that can induce a neuron to switch between class I and II excitabilities are from external influences outside the neuron. However, there are only a few^32^ investigations on the switch between class I and II excitabilities induced by an important self-modulation to the current across the membrane–autapse^33^. Autapse of a neuron is a special synapse that connects to the neuron itself^33^, and has been observed anatomically in nervous systems such as in the cerebral cortex, neocortex, cerebellum, hippocampus, striatum, and visual cortex^33–39^. Through biological experiments^34–36^, autapses have been found to play many roles, such as in the persistent firings induced by positive self-feedback mediated by excitatory autapses^34^, and repetitive firing with precise spike timing induced by inhibitory autapses^35, 36^. Model simulations also found that autapses have other roles in neuronal systems^32, 40–61^, for example, autapse-induced stochastic resonance, coherence resonance, and formations in isolated neurons or neuronal networks. A recent study^32^ shows that inhibitory autapse can induce interneuron switches from class I excitability to class II excitability/spiking characterized by codimension-1 bifurcations and frequency responses. Inhibitory and excitatory autapses of a neuron can generate “negative” autaptic current and “positive” autaptic current, respectively, i.e., an outward current and inward current, to the neuron itself. The current mediated by the autapse may induce transition between the 2 classes of excitability through changes in competition between outward and inward currents, the position relationship between nullclines, bifurcations, as well as firing frequency.

In the present paper, we proposed a comprehensive viewpoint that autapses can induce transition between class I and II excitabilities/spikings in the ML model. That is, inhibitory autapses can induce transition from class I to II excitability due to the outward current mediated by the autapse, wherein the role of autaptic current is identified as the M-type potassium (*K* ^+^) current. Excitatory autapses can induce transition from class II to class I spiking because of the inward current mediated by the autapse, and the role of autaptic current is the same as that of the L-type potassium (*Ca* ^2+^) current. In this paper, we described the transitions between class I and II excitabilities/spikings by firing frequency responses, net current at steady-state, and different bifurcations. Except the well-known SNIC corresponding to class I excitability/spiking and subcritical Hopf bifurcation to class II excitability, more complex codemension-1 and -2 bifurcations related to excitability/spiking classes were identified. The results of the present work not only provided a novel function of the inhibitory autapse in regulating neuronal firing dynamics, which is helpful for understanding the roles of different kinds of autapses in neuroscience and neurodynamics, but also presented a comprehensive relationship between the classes of neuronal excitability/spiking and bifurcations, firing frequency responses, current-potential curves at steady-state, and nullclines. Different from the investigation^32^, the codimension-2 bifurcations and biophysical basis of the transition between classes of neuronal excitability and the excitatory autapse were investigated in the present study.

## Results

### The influence of the inhibitory and excitatory autapses on the nullcline $\mathop{V}\limits^{.}=0$

The introduction of the autapse to the ML model changed the nullcline $\dot{V}=0$, and therefore changed the position relationship between $\dot{V}=0$ and $\dot{w}=0$, which provided the possibility of bifurcations changes and a transition between excitability/spiking classes. This is because the position relationship between $\dot{V}=0$ and $\dot{w}=0$ determined the bifurcations.

**Figure 1:**
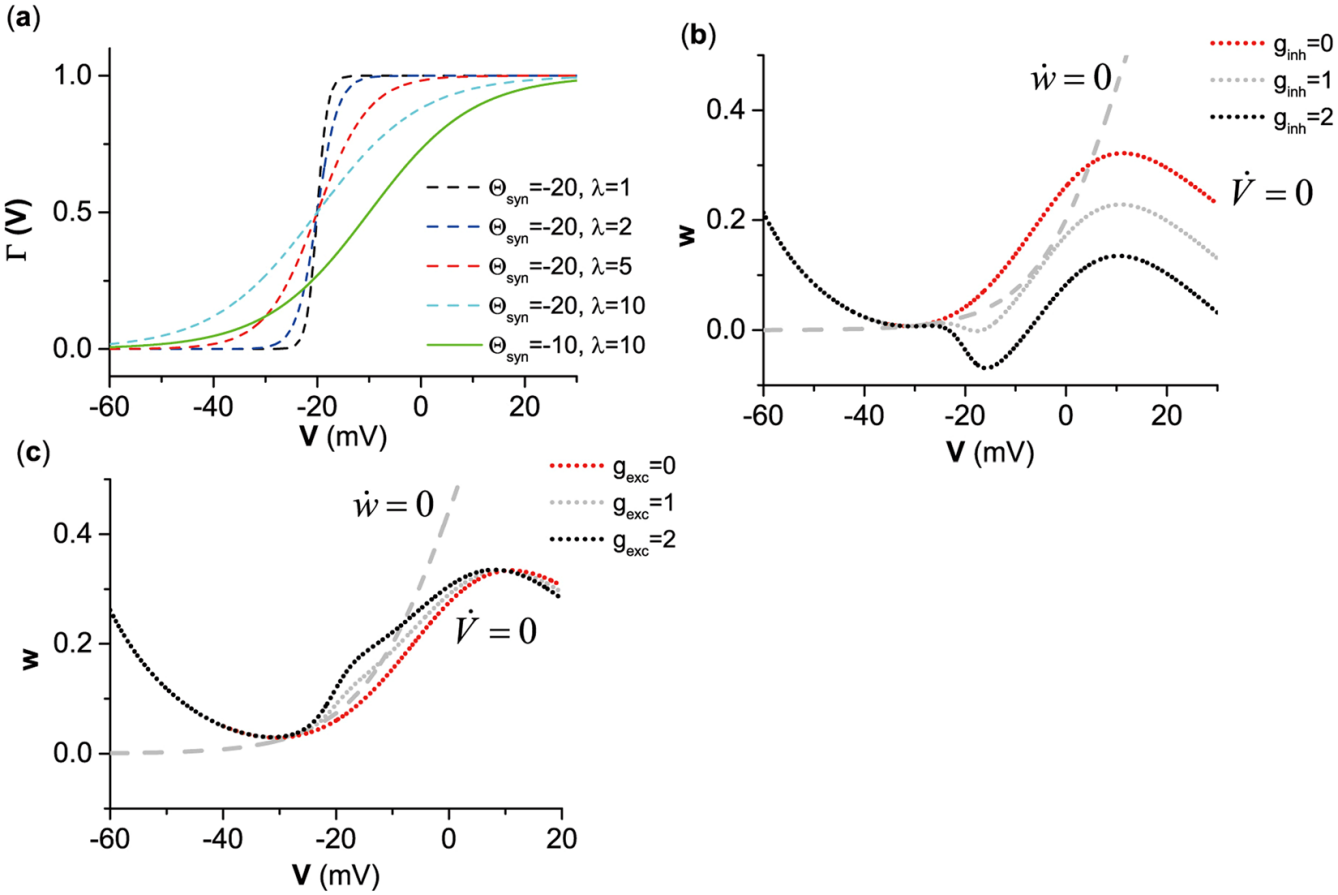
Effects of autaptic currents on nullcline $\dot{V}=0$. (**a**) The curves of Γ(*V*) with different Θ_*syn*_ and *λ*. (**b**) Changes of the nullcline $\dot{V}=0$ induced by the inhibitory autapse and *I*_*app*_ = 39.96 *μ*A/cm^2^. (**c**) Changes of the nullcline $\dot{V}=0$ induced by the excitatory autapse and *I*_*app*_ = 52.765 *μ*A/cm^2^. In (**b**,**c**), the gray dashed curve represents nullcline of $\dot{w}=0$, the dotted curve with different colors represent nullcline $\dot{V}=0$ at different autaptic strengths.

The function Γ(*V* − Θ_*syn*_) = 1/$(1+exp(-(V-{{\rm{\Theta }}}_{syn})/\lambda ))$ in the autapse model ${I}_{aut}={g}_{aut}(V-{V}_{syn})\,{\rm{\Gamma }}(V-{{\rm{\Theta }}}_{syn})$ is shown in Fig. 1(a), which implies that autaptic current in the present study (Θ_*syn*_ = −20 mV, *λ* = 1, 2) took effect in the range of *V* > −30 mV. As *g*_*inh*_ (*g*_*exc*_) increased, the curve shape of $\dot{V}=0$ exhibited 2 characteristics when *I*_*app*_ was fixed at *I*_*app*_ = 39.96 *m*A/cm^2^ and *I*_*app*_ = 52.765 *m*A/cm^2^, respectively, as shown in Fig. 1(b,c). The first is that the part when *V* > −30 mV shifts down for the inhibitory autapses and up for the excitatory autapses. The other characteristic is that the shape of nullcline $\dot{V}=0$ around *V* = −30 mV changes for both inhibitory and excitatory autapses, as shown in Fig. 1(b,c). As *I*_*app*_ increased or decreased, the nullcline $\dot{V}=0$ moved up or down, leading to changes in the position relationship between $\dot{V}=0$ and $\dot{w}=0$, which implied that transitions between bifurcations could be induced by changes in *g*_*inh*_ (*g*_*exc*_) and *I*_*app*_.

### Notations of nonlinear dynamics or bifurcations

For convenience, notations about codim-1 and codim-2 bifurcations encountered in the present paper are summarized in Table 1.

**Table 1:** Notations and abbreviations of the bifurcations.

Co-dimension	Bifurcations	Abbreviations
Codim-1	Supercritical Hopf bifurcation	SupH
	Saddle node bifurcation	${{\rm{SN}}}_{{\rm{i}}}^{{\rm{S}}}$ or ${{\rm{SN}}}_{{\rm{i}}}^{{\rm{U}}}$, i = 1, 2
	Saddle node on invariant circle	SNIC
	Saddle homoclinic orbit	BHom and SHom
	Saddle node bifurcation of limit cycle	LPC
Codim-2	Cusp bifurcation	CP
	Bautin bifurcation	GH
	Bogdanov-Takens bifurcation	BT
	saddle-node separatrix-loop	SNHO

For codim-1 bifurcations, SubH and SupH respectively represent subcritical and supercritical Hopf bifurcations. ${{\rm{SN}}}_{{\rm{i}}}^{{\rm{S}}}$ and ${{\rm{SN}}}_{{\rm{i}}}^{{\rm{U}}}$ are the saddle-node bifurcation of equilibrium, where the superscript “S” (“U”) means that a stable (unstable) node collides with a saddle, and the subscript “i” is an integer to indicate the sequence of the appearance of this kind of bifurcation. SNIC is saddle-node on invariant circle bifurcation. BHom and SHom represent saddle homoclinic bifurcation, where “B” and “S” are used to distinguish the size of the saddle homoclinic orbit. LPC is saddle-node (fold) bifurcation of the limit cycle.

For codim-2 bifurcations, CP represents cusp bifurcations of equilibrium, GH is Bautin (degenerate Hopf) bifurcations of equilibrium, BT represents Bogdanov-Takens bifurcations of equilibrium, and SNHO is a saddle-node separatrix-loop.

### Transition from class I to class II excitability/spiking induced by the inhibitory autapse

In this subsection, the ML model without inhibitory autapse exhibited class I excitability/spiking. We investigated the dynamics of the ML model after the introduction of an inhibitory autapse.

#### Six examples of one-parameter bifurcations and firing frequency responses

In the ML model with and without inhibitory autapses, 6 different cases of bifurcations related to excitability/spiking and transition from class I to class II excitability/spiking were identified, as shown in Table 2. Six representative examples corresponding to the 6 cases are introduced here in details.

**Table 2:** Six cases of bifurcations corresponding to excitability/spiking classes induced by inhibitory autapse.

Case	*g*_*inh*_	Resting state → Spiking (*I*_*app*_ increases)			Spiking → Resting state (*I*_*app*_ decreases)		
		Bifurcation	Frequency	Excitability	Bifurcation	Frequency	Spiking
1	(0, 0.3645)	SNIC	Zero	I	SNIC	Zero	I
2	(0.3645, 0.385)	${{\rm{SN}}}_{{\rm{4}}}^{{\rm{S}}}$	Nonzero	II	SNIC	Zero	I
3	(0.385, 0.55)	${{\rm{SN}}}_{{\rm{4}}}^{{\rm{S}}}$	Nonzero	II	BHom	Zero	I
4	(0.55, 3.032)	SubH	Nonzero	II	BHom	Zero	I
5	(3.032, 4.05)	SubH	Nonzero	II	LPC	Nonzero	II
6	(4.05, 5.0)	SupH	Nonzero	II	SupH	Nonzero	II

**Figure 2:**
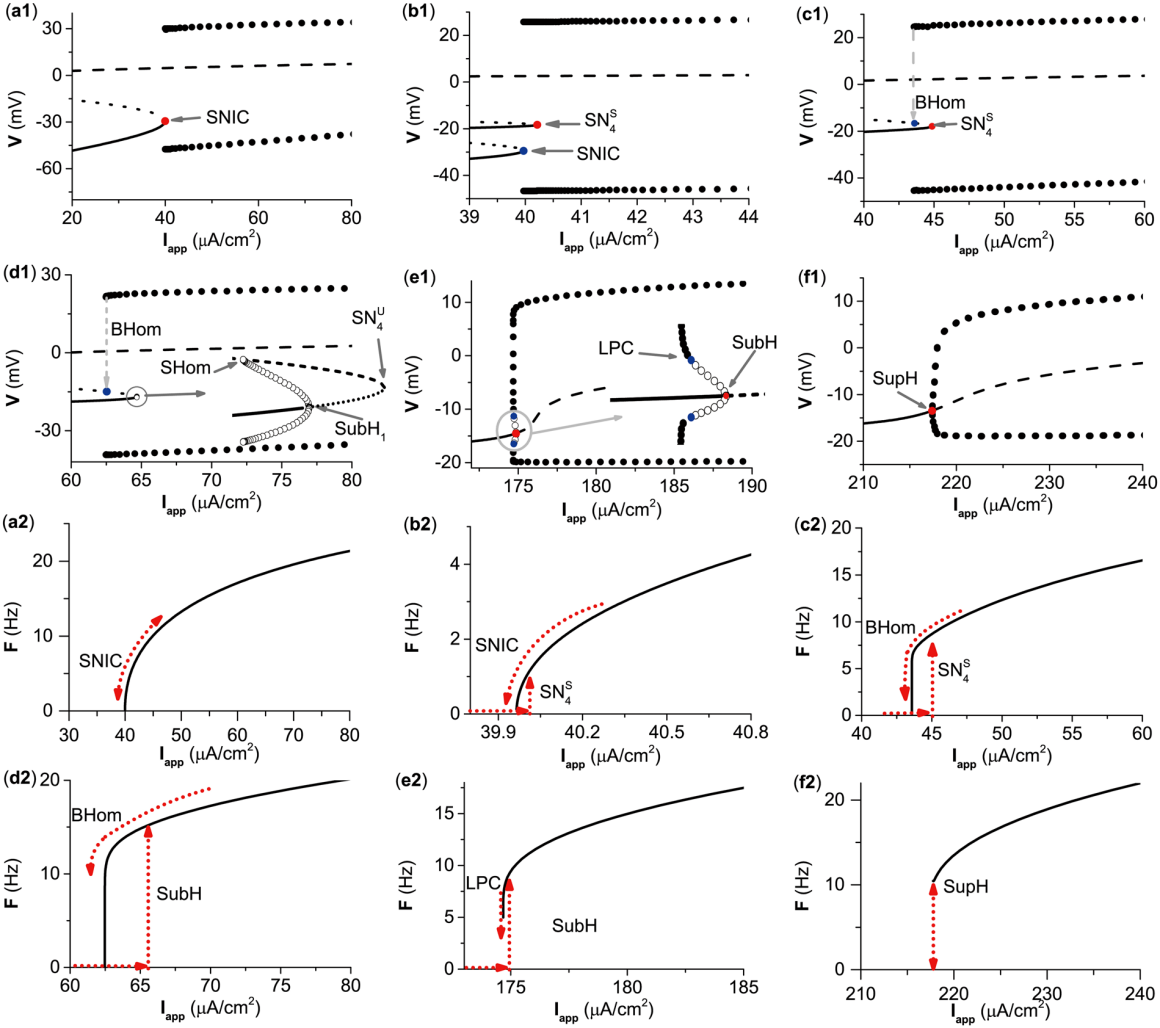
Dynamics of the ML model with respect to *I*_*app*_ at different *g*_*exc*_ values. (**a1**–**f1**) Bifurcation diagrams showing voltage at equilibriums and at max/min of limit cycle as *I*_*app*_ is increased when *g*_*inh*_ at different values. (**a2**–**f2**) Frequency-current (F-I) curves showing firing frequency as *I*_*app*_ is increased when *g*_*inh*_ at different values. (**a1**,**a2**) *g*_*inh*_ = 0 *μ*S/cm^2^. (**b1**,**b2**) *g*_*inh*_ = 0.372 *μ*S/cm^2^. (**c1**,**c2**) *g*_*inh*_ = 0.5 *μ*S/cm^2^. (**d1**,**d2**) *g*_*inh*_ = 1.0 *μ*S/cm^2^. (**e1**,**e2**) *g*_*inh*_ = 3.5 *μ*S/cm^2^. (**f1**,**f2**) *g*_*inh*_ = 4.4 *μ*S/cm^2^. In panels (a1–f1), the solid, dotted and dashed lines represent stable equilibrium, saddle and unstable equilibrium, respectively. The upper and lower solid (open) circles are the maximum and minimum values of the stable (unstable) limit cycle, respectively. SNIC represents saddle-node bifurcation on an invariant circle. SubH and SupH represent subcritical Hopf bifurcation and supercritical Hopf bifurcation, respectively. BHom and SHom represent saddle homoclinic orbit bifurcation. ${{\rm{SN}}}_{{\rm{4}}}^{{\rm{S}}}$ and ${{\rm{SN}}}_{{\rm{4}}}^{{\rm{U}}}$ represent saddle node bifurcation of equilibrium. LPC represents saddle node bifurcation of limit cycle.

The 6 examples of the bifurcation and firing frequency response with *g*_*inh*_ = 0, 0.372, 0.5, 1.0, 3.5, and 4.4 *μ*S/cm^2^ are shown in Fig. 2. For case 1 and case 6, changes from the resting state to spiking (and vice versa) were through the same bifurcation point, and no coexisting behaviors were detected. For cases 2 to 5, the bifurcation via which the resting state changes to spiking was different from the bifurcation from the spiking to resting state showed the coexistence of a resting state and spiking. For case 1 to case 6, the bifurcations related to excitability classes, corresponding to bifurcation from the resting state to spiking, were SNIC (*I*_*app*_ ≈ 39.96 *μ*A/cm^2^), ${{\rm{SN}}}_{4}^{{\rm{S}}}$ (*I*_*app*_ ≈ 40.21 *μ*A/cm^2^), ${{\rm{SN}}}_{{\rm{4}}}^{{\rm{S}}}$ (*I*_*app*_ ≈ 44.85 *m*A/cm^2^), SubH (*I*_*app*_ ≈ 64.67 *m*A/cm^2^), SubH (*I*_*app*_ ≈ 174.85 *m*A/cm^2^), and SupH (*I*_*app*_ ≈ 217.39 *m*A/cm^2^), respectively; meanwhile, bifurcations associated with the spiking classes, corresponding to bifurcation from the spiking to resting state, were SNIC (*I*_*app*_ ≈ 39.96 *μ*A/cm^2^), SNIC (*I*_*app*_ ≈ 39.96 *μ*A/cm^2^), BHom (*I*_*app*_ ≈ 43.57 *μ*A/cm^2^), BHom (*I*_*app*_ ≈ 62.49 *μ*A/cm^2^), LPC (*I*_*app*_ ≈ 174.71 *μ*A/cm^2^), and SupH (*I*_*app*_ ≈ 217.39 *μ*A/cm^2^), respectively.

For case 1, as *I*_*app*_ approached the SNIC, the firing frequency exhibited a nearly 0 value, corresponding to the infinite period of the invariant cycle, as depicted in Fig. 2(a2), which shows that the excitability/spiking was class I. For cases from 2 to 6, the firing frequency exhibited a non-zero value as *I*_*app*_ increased across the bifurcation point from the resting state to spiking, showing that the excitability was class II (Fig. 2(b–f)). Furthermore, the bifurcation SNIC was related to class I excitability, and bifurcations SN, SubH, and SupH were related to class II excitability. Transition from class I excitability to class II excitability occurred when case 1 changed to case 2. It should be emphasized that 2 stable nodes corresponding to the resting state appeared in case 2. One was related to the bifurcation SNIC appearing at *I*_*app*_ ≈ 39.96 *μ*A/cm^2^, while the other corresponded to the ${{\rm{SN}}}_{{\rm{4}}}^{{\rm{S}}}$ located at *I*_*app*_ ≈ 40.21 *m*A/cm^2^, as shown in Fig. 2(b1). Since the bifurcation ${{\rm{SN}}}_{{\rm{4}}}^{{\rm{S}}}$ was located right next to the SNIC, the ${{\rm{SN}}}_{{\rm{4}}}^{{\rm{S}}}$, across which the resting state changed to spiking, is suggested to be related to the excitability class in case 2.

Additionally, the class of spiking in case 2 was still related to SNIC and still class I, as depicted in Fig. 2(b2). For case 3 and case 4, the firing frequency exhibited a nearly 0 value as the spiking changed to the resting state via the BHom, which shows that the spiking was still class I (Fig. 2(c2–d2)). Different from the SNIC (case 1), wherein the firing frequency increased relative slowly with increasing *I*_*app*_, the firing frequency exhibited a fast increase with a logarithmic scale near the BHom^2^. For case 5 and case 6, the firing frequency exhibited a non-zero value as *I*_*app*_ decreased across the bifurcation point from the spiking to resting state, thus showing that the spiking was class II (Fig. 2(e2–f2)). Moreover, the transition from class I spiking to class II spiking appeared when case 4 changed to the case 5. The SNIC and BHom were associated with class I spiking, while LPC and SupH corresponded to class II spiking. With decreasing across the LPC, a stable limit cycle and an unstable limit cycle collided with each other and disappeared, as shown in Fig. 2(f1). The stable limit cycle exhibited a non-zero firing frequency. For example, the firing frequency was 7.5 Hz (non-zero) when *I*_*app*_ = 174.72 *μ*A/cm^2^, as shown in Fig. 2(f2).

#### Position relationship between nullclines, dynamics in the phase plane, and firing frequency near the bifurcation point

**Figure 3:**
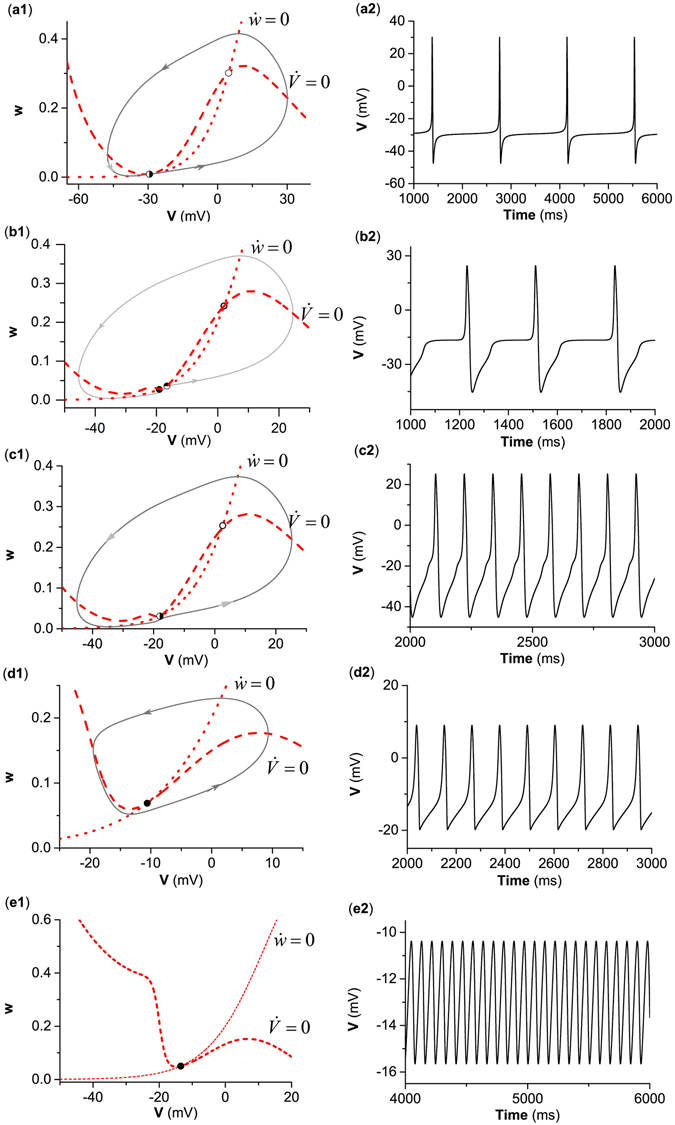
Dynamics corresponding to or near the different bifurcation points. (**a**) SNIC with *g*_*inh*_ = 0 *μ*S/cm^2^; (**b**) BHom with *g*_*inh*_ = 0.5 *μ*S/cm^2^; (**c**) ${{\rm{SN}}}_{{\rm{4}}}^{{\rm{S}}}$ with *g*_*inh*_ = 0.5 *μ*S/cm^2^; (**d**) SubH with *g*_*inh*_ = 3.5 *μ*S/cm^2^; (**e**) SupH at = *g*_*inh*_ = 4.4 *μ*S/cm^2^. Left: nullclines, equilibriums, and limit cycles or orbits in the phase portraits; Right: Spike trains. Parameter values: (**a2**) *I*_*app*_ = 39.98 *μ*A/cm^2^; (**b2**) *I*_*app*_ = 43.572 *μ*A/cm^2^; (**c2**) *I*_*app*_ = 44.8461 *μ*A/cm^2^; (**d2**) *I*_*app*_ = 179.2 *μ*A/cm^2^; (**e2**) *I*_*app*_ = 217.39 *μ*A/cm^2^. In panels (a1–e1), the red dotted line and dashed line represent nullcline $\dot{w}=0$ and nullcline $\dot{V}=0$, respectively. The solid line represents trajectory and arrow indicates direction.

The SNIC is a standard saddle-node bifurcation point located on an invariant circle with an infinite period. The saddle-node point (half hollow circle) and the invariant circle (gray solid line) for the SNIC (Fig. 2(a1)) are shown in Fig. 3(a1). The saddle-node bifurcation point corresponds to the tangent point (−29.39, 0.0085) between nullclines $\dot{V}=0$ (red dashed curve) and $\dot{w}=0$ (red dotted curve) in the phase plane. As *I*_*app*_ approached the SNIC point (*I*_*app*_ ≈ 39.96 *μ*A/cm^2^), the spiking exhibited a long period, i.e., a low firing frequency because of being near the SNIC. For example, the spiking frequency with (*I*_*app*_ = 39.97 *μ*A/cm^2^) was very low (0.72 Hz, Fig. 3(a2)). This is because that SNIC corresponded to class I excitability/spiking.

The BHom corresponds to a saddle contacted with a stable limit cycle, which is a homoclinic orbit with an infinite period. If the limit cycle is unstable, the saddle homoclinic bifurcation is subcrtical. For the BHom shown in Fig. 2(c1), the saddle (half hollow circle) is the intersection points between the nullclines $\dot{V}=0$ (red dashed curve) and $\dot{w}=0$ (red dotted curve), as shown in Fig. 3(b1). The homoclinic orbit is shown by the gray curve. In addition, a stable node and an unstable focus are shown by the solid black point and the hollow circle, respectively. If a spiking was close to the BHom (*I*_*app*_ ≈ 43.57 *μ*A/cm^2^), the spiking also exhibited a low firing frequency due to the homoclinic orbit. For example, when *I*_*app*_ = 43.57 *μ*A/cm^2^, the spiking exhibited a low frequency (3.3 Hz), as shown in Fig. 3(b2). This is because that BHom was related to class I spiking.

The ${{\rm{SN}}}_{{\rm{4}}}^{{\rm{S}}}$ (*I*_*app*_ ≈ 44.8461 *μ*A/cm^2^) shown in Fig. 2(c1) corresponds to the tangent point (−17.936, 0.031) (half hollow circle) between the nullclines $\dot{V}=0$ (red dashed curve) and $\dot{w}=0$ (red dotted curve), which is a standard saddle-node bifurcation ***off*** limit cycle, as illustrated in Fig. 3(c1). When *I*_*app*_ = 40.21 *μ*A/cm^2^, the spiking exhibited non-zero frequency (8.55 Hz) (Fig. 3(c2)). This is because that ${{\rm{SN}}}_{{\rm{4}}}^{{\rm{S}}}$ was associated with class II excitability.

For SubH (*I*_*app*_ ≈ 174.85 *μ*A/cm^2^) shown in Fig. 2(d), the equilibrium corresponds to the intersection point (−10.59, 0.069, solid dot) between nullclines $\dot{V}=0$ (red dashed curve) and $\dot{w}=0$ (red dotted curve), as shown in Fig. 3(d1). In addition, a spiking (gray solid curve) appeared at *I*_*app*_ = 174.85 *μ*A/cm^2^. This spiking was a limit cycle bifurcated from the LPC that appeared at *I*_*app*_ = 174.71 *μ*A/cm^2^ and near the SubH. When *I*_*app*_ = 174.85 *μ*A/cm^2^, the spiking exhibited a fixed non-zero firing frequency (8.82 Hz) (Fig. 3(d2)). This is because that SubH was associated with class II excitability.

SupH (*I*_*app*_ ≈ 217.3 *μ*A/cm^2^) shown in Fig. 2(e) corresponds to the intersection point (−13.481, 0.0507) (the solid dot) between nullclines $\dot{V}=0$ (red dashed curve) and $\dot{w}=0$ (red dotted curve) in the phase plane, as shown in Fig. 3(e1). The amplitude of the limit cycle increased from 0 as *I*_*app*_ increased, i.e., there was a transition from subthreshold oscillation to spiking with nearly fixed, non-zero firing frequency. For example, when *I*_*app*_ = 217.6 *m*A/cm^2^, the neuron exhibited a fast subthreshold oscillation (12 Hz) (Fig. 3(e2)). This is because that SupH was associated with class II excitability.

#### Steady-state I–V curves for 5 types of codimension-1 bifurcations

In previous studies for SNIC and SubH, ∂*I*/∂*V* = 0 and ∂*I*/∂*V* < 0, respectively, were used to characterize class I and II excitability. In fact, the inhibitory autapse could induce changes in I–V curves because $I(V)={C}^{-1}(-{I}_{Ca}(V)-{I}_{K}(V)-{I}_{L}(V)-{I}_{aut}(V))$.

**Figure 4:**
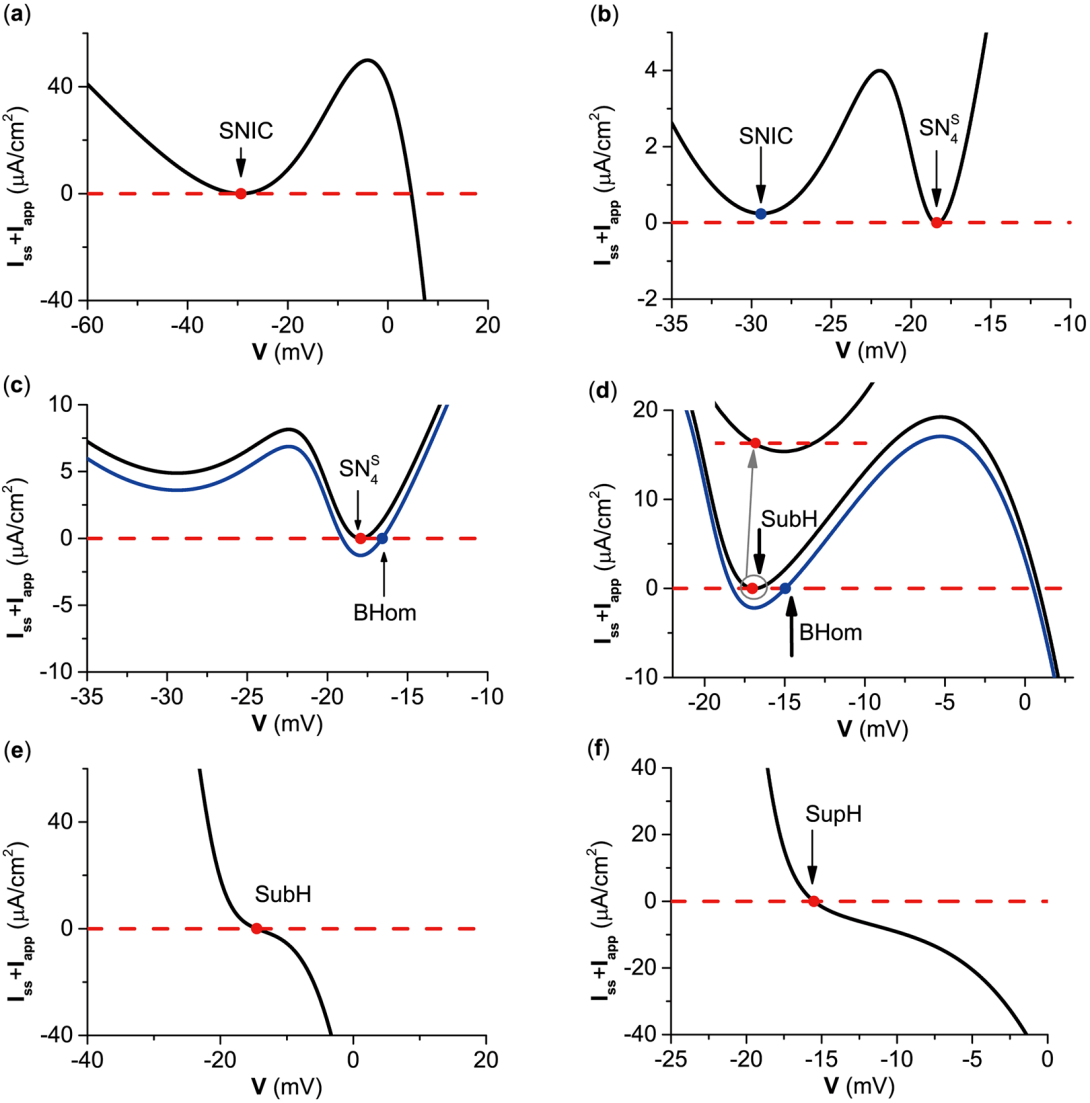
Steady state I–V curves for 5 bifurcation points related to equilibrium. (**a**) *g*_*inh*_ = 0 *μ*S/cm^2^; (**b**) *g*_*inh*_ = 0.372 *μ*S/cm^2^; (**c**) *g*_*inh*_ = 0.5 *μ*S/cm^2^; (**d**) *g*_*inh*_ = 1.0 *μ*S/cm^2^; (**e**) *g*_*inh*_ = 3.5 *μ*S/cm^2^; (**f**) *g*_*inh*_ = 4.4 *μ*S/cm^2^. SNIC represents saddle-node bifurcation on an invariant circle. SubH and SupH represent subcritical Hopf bifurcation and supercritical Hopf bifurcation, respectively. BHom represents saddle homoclinic orbit bifurcation. ${{\rm{SN}}}_{{\rm{4}}}^{{\rm{S}}}$ represents saddle node bifurcation of equilibrium.

In the present paper, the I–V curves for 5 types of codimension-1 bifurcations related to equilibrium point corresponding to Fig. 1(a–f) are respectively shown in Fig. 4(a–f). For SNIC and ${{\rm{SN}}}_{{\rm{4}}}^{{\rm{S}}}$, ∂*I*/∂*V* = 0, which corresponds to the net perithreshold current at steady-state being inward. Meanwhile, ∂*I*/∂*V* < 0 for SubH and SupH, which shows that current at steady-state monotonically decreased with respect to the increase in *V*, and the net perithreshold current at steady-state was outward. For BHom, ∂*I*/∂*V* > 0, which corresponds to the net perithreshold current at steady-state being inward. Investigations for the SNIC and SubH can can be found in the literature^21^, while the I–V curves for saddle-node bifurcations, SupH, and BHom are new findings. ∂*I*/∂*V* = 0, ∂*I*/∂*V* < 0, and ∂*I*/∂*V* > 0 were proved as the necessary conditions for SNIC and SN bifurcations, SubH and SupH bifurcations, and for BHom bifurcations, respectively. The detailed proofs are provided in the Methods section.

#### Codimension-2 bifurcations related to transitions from class I to II excitability/spiking

**Figure 5:**
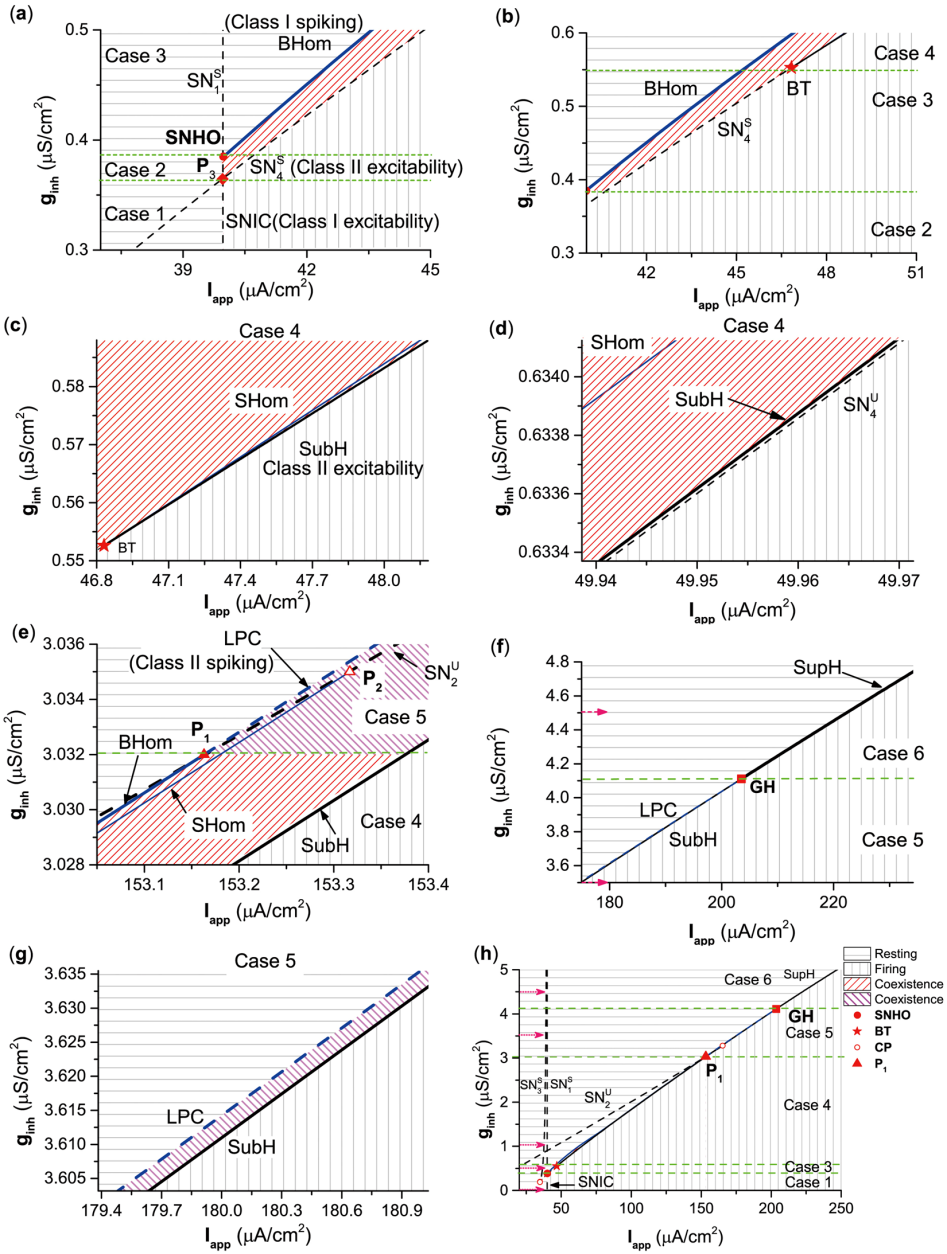
Two-parameter bifurcations in the plane (*I*_*app*_, *g*_*inh*_) of the ML model with an inhibitory autapse. (**a**) Partial enlargement of the bifurcation diagram around SNHO and *P*_3_. (**b**) Partial enlargement of the bifurcation diagram around BT. (**c**) Partial enlargement of the bifurcation diagram is up and to the right of BT in (**b**). (**d**) Further amplification is up and to the right of BT in (**b**). (**e**) Partial enlargement of the bifurcation diagram around *P*_1_. (**f**) Partial enlargement of the bifurcation diagram around GH in (**h**). (**g**) Partial enlargement of the bifurcation diagram is up and to the right of GH. (**h**) Global view of the bifurcation diagram. Figure (**a**–**f**) is a part of Fig. (**h**). SNIC represents the curve of saddle-node bifurcation on an invariant circle. SubH and SupH are subcritical Hopf curve and supercritical Hopf curve, respectively. BHom and SHom are saddle homoclinic orbit bifurcation curves. ${{\rm{SN}}}_{{\rm{i}}}^{{\rm{S}}}$ and ${{\rm{SN}}}_{{\rm{i}}}^{{\rm{U}}}$ (*i* = 1, 2, 3, 4) are curves of saddle node bifurcation of equilibrium. LPC represents the curve of saddle node bifurcation of limit cycle. CP is cusp bifurcation. BT is Bogdanov-Takens bifurcation. SNHO is saddle-node separatrix-loop. GH is Bautin bifurcation.

We then investigated the bifurcation diagrams in the plane (*I*_*app*_, *g*_*inh*_), and acquired comprehensive views of transitions from class I to class II excitability/spiking (Fig. 5). The detailed dynamics of special or codimension-2 bifurcation points and codimension-1 bifurcation curves related to class I to class II excitability/spiking are introduced firstly, and then the whole framework of the bifurcations in the plane (*I*_*app*_, *g*_*inh*_) are provided. The resting state, coexisting behaviors of the resting and spiking states, and spiking state, are labeled with gray horizontal lines, red or pink sloping lines, and gray vertical lines, respectively.

The borders between 2 neighboring cases from cases 1 to 6 are horizontal green lines across 5 special points, *P*_3_, SNHO, BT, *P*_1_, and GH, with increasing *g*_*inh*_. *P*_3_ (*I*_*app*_ ≈ 36.96 *μ*A/cm^2^, *g*_*inh*_ ≈ 0.3645 *μ*S/cm^2^) is the intersection point between the bifurcation curves ${{\rm{SN}}}_{{\rm{4}}}^{{\rm{S}}}$ and SNIC, as shown in Fig. 5(a). The SNHO (red solid circle) (*I*_*app*_ ≈ 39.98 *μ*A/cm^2^, *g*_*inh*_ ≈ 0.385 *μ*S/cm^2^) is the intersection point between the SNIC curve and ${{\rm{SN}}}_{{\rm{1}}}^{{\rm{S}}}$ curve, and is the origin of the BHom curve (Fig. 5(a)). The BT (red star) point (*I*_*app*_ ≈ 46.83 *μ*A/cm^2^, *g*_*inh*_ ≈ 0.55 *μ*S/cm^2^) corresponds to the tangent point between the curves of SubH and saddle-node bifurcations (${{\rm{SN}}}_{{\rm{4}}}^{{\rm{S}}}$ and ${{\rm{SN}}}_{{\rm{4}}}^{{\rm{U}}}$), and is the origin of the SHom curve, as shown in Fig. 5(b,c). The position relationship between the 3 bifurcation curves, SubH, SHom, and ${{\rm{SN}}}_{{\rm{4}}}^{{\rm{U}}}$ are shown in Fig. 5(d). The *P*_1_ point (*I*_*app*_ ≈ 151.163 *μ*A/cm^2^, *g*_*inh*_ ≈ 3.032 *μ*S/cm^2^) is the termination of the bifurcation curve BHom and the origin of the LPC curve, and is located on the saddle-node bifurcation curve of ${{\rm{SN}}}_{{\rm{2}}}^{{\rm{U}}}$, as shown in Fig. 5(e). The GH point (*I*_*app*_ ≈ 203.55 *μ*A/cm^2^, *g*_*inh*_ ≈ 4.11 *μ*S/cm^2^) is the transition point between curves SubH and SupH, and is the termination of the LPC curve, as shown in Fig. 5(f). The position relationship between bifurcation curves SubH and LPC are shown in Fig. 5(g).

Bifurcation curves SNIC, ${{\rm{SN}}}_{{\rm{4}}}^{{\rm{U}}}$, SubH, and SupH, and bifurcation/special points *P*_3_, BT, and GH are related to excitability classes. Right next to these curves, the behavior is spiking. The SNIC curve was related to class I excitability, while the other curves were associated with class II excitability. The transition from class I to class II excitabilities occurred at point *P*_3_.

Bifurcation curves SNIC, BHom, LPC, and SupH, and special bifurcation points *P*_3_, SNHO, *P*_1_, and GH are related to spiking classes. To the left of these curves, the behavior is resting. The SNIC and BHom curves corresponded to class I spiking, while the LPC and SupH curves were related to class II spiking. The transition from class I to class II spiking occurred at point *P*_1_.

The framework of the dynamic behaviors, codimension-2 points, and bifurcation curves are shown in Fig. 5(h). The different codimension-1 bifurcation curves ran from bottom-left to upper-right to form the borders between resting and firing states. The 5 points, *P*_3_, SNHO, BT, *P*_1_, and GH, are shown by the red rhombus, red solid circle, red star, red triangle, and red solid square, respectively. The horizontal green lines run across the 5 points to form the border between the 6 cases, and the 6 pink arrows with dotted lines represent the positions of the 6 examples of bifurcations. However, the parameter region of the coexisting behaviors was too small to be visible in the scale used in Fig. 5(h). In addition, there were other codimension-1 bifurcation curves (${{\rm{SN}}}_{{\rm{3}}}^{{\rm{S}}}$ and ${{\rm{SN}}}_{{\rm{2}}}^{{\rm{U}}}$) and bifurcation points (*P*_2_ and CP), that had no direct relationship to the transition between neuronal excitability/spiking classes, and thus were not introduced in the present paper.

### Transition from class II to class I spiking and the bifurcations induced by the excitatory autapse

In this subsection, we discuss how the ML model without excitatory autapses exhibited class II excitability/spiking. Then, we present the dynamics of the ML.

#### Three cases of one-parameter bifurcations and firing frequency responses

**Figure 6:**
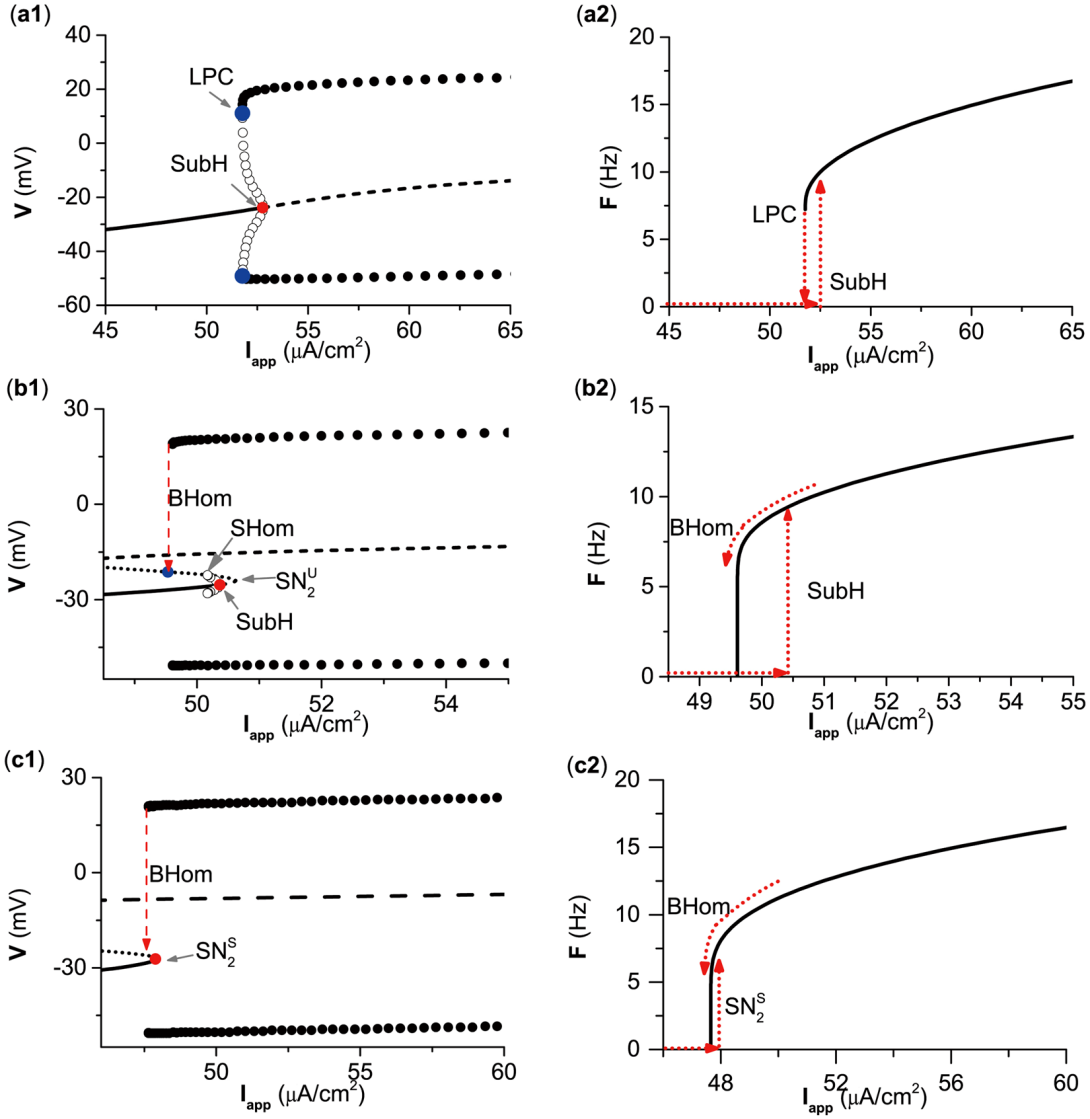
Dynamics of the ML model with respect to *I*_*app*_ at different *g*_*exc*_ values. (**a**) *g*_*exc*_ = 0.0 *μ*S/cm^2^; (**b**) *g*_*exc*_ = 0.5 *μ*S/cm^2^; (**c**) *g*_*exc*_ = 2.0 *μ*S/cm^2^. Left: bifurcation diagrams of *V*; Right: firing frequency-current curve. In panels (a1–c1), the solid, dotted and dashed lines represent stable equilibrium, saddle and unstable equilibrium, respectively. The upper and lower solid (open) circles are the maximum and minimum values of the stable (unstable) limit cycle, respectively. BHom and SHom represent saddle homoclinic orbit bifurcation. ${{\rm{SN}}}_{{\rm{2}}}^{{\rm{S}}}$ represents saddle node bifurcation of equilibrium. LPC represents saddle node bifurcation of limit cycle.

We identified 3 cases of bifurcations related to classes of excitability/spiking, as shown in Table 3, and the corresponding examples (*g*_*exc*_ = 0, 0.5 and 2.0 *μ*S/cm^2^) are shown in Fig. 6. All 3 cases exhibited coexisting behaviors lying between the resting state and spiking.

**Table 3:** Three cases of bifurcations corresponding to neuronal excitability/spiking classes induced by the excitatory autapse.

Case	*g*_*exc*_	Resting state → Spiking (*I*_*app*_ increases)			Spiking → Resting state (*I*_*app*_ decreases)		
		Bifurcation	Frequency	Excitability	Bifurcation	Frequency	Spiking
1	(0, 0. 406)	SubH	Nonzero	II	SubH	Nonzero	II
2	(0.406, 1.59)	SubH	Nonzero	II	BHom	Zero	I
3	(1.5977, 2.0)	${{\rm{SN}}}_{{\rm{2}}}^{{\rm{S}}}$	Nonzero	II	BHom	Zero	I

For cases 1 to 3, the bifurcations related to the excitability class were SubH (*I*_*app*_ ≈ 52.765 *μ*A/cm^2^), SubH (*I*_*app*_ ≈ 50.36 *μ*A/cm^2^) and ${{\rm{SN}}}_{{\rm{2}}}^{{\rm{S}}}$ (*I*_*app*_ ≈ 47.9 *μ*A/cm^2^). All 3 cases exhibited class II excitability. The bifurcations associated with the spiking class were LPC (*I*_*app*_ ≈ 51.75 *μ*A/cm^2^), BHom (*I*_*app*_ ≈ 49.6 *μ*A/cm^2^), and BHom (*I*_*app*_ ≈ 47.66 *μ*A/cm^2^) for cases 1, 2, and 3, respectively. Case 1 exhibited class I spiking, while case 2 and case 3 had class II spiking. The transition from class I spiking to class II spiking occurred when case 1 changed to case 2.

#### The steady I–V curves for 5 types of codimension-1 bifurcations

**Figure 7:**
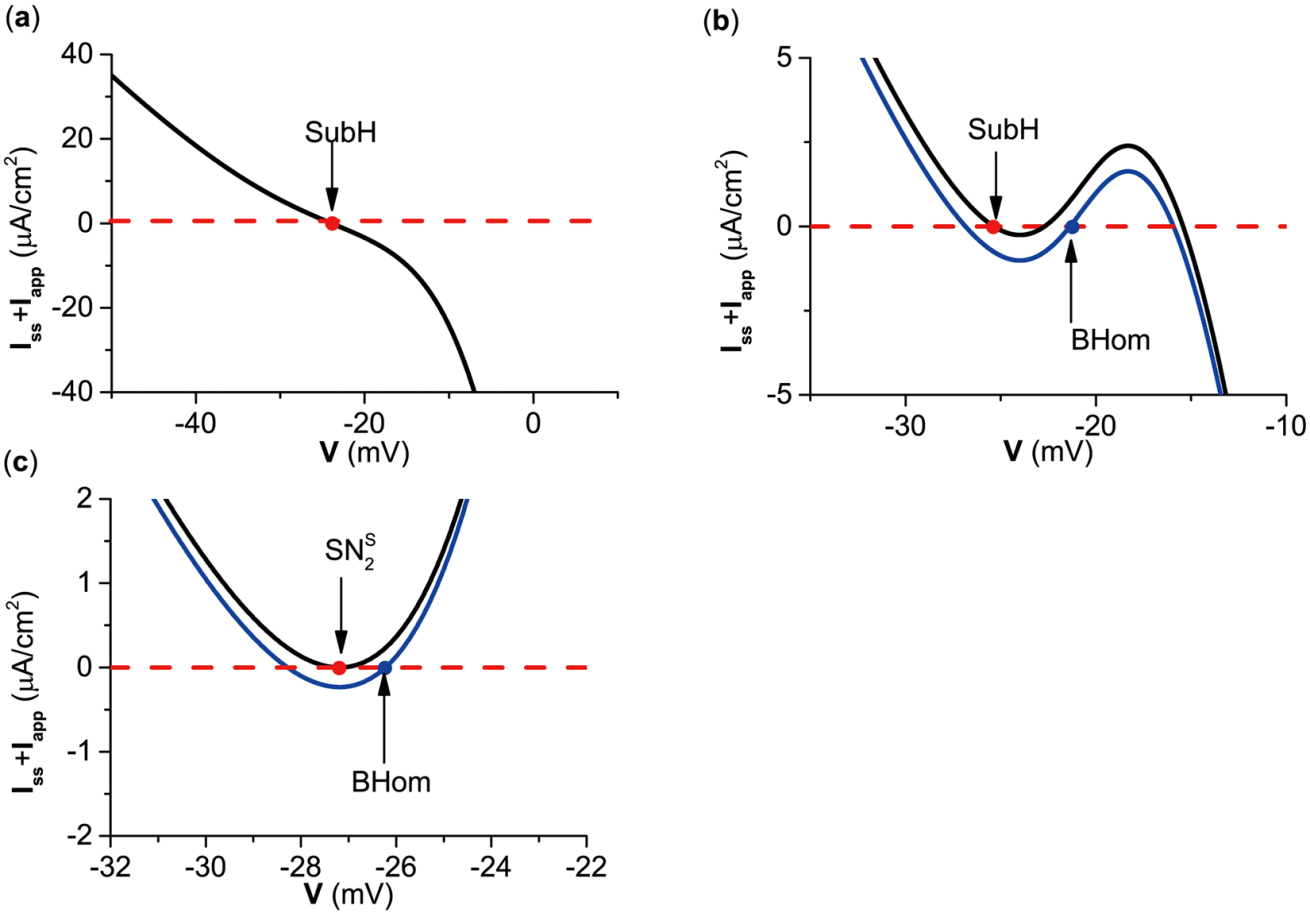
Steady state I–V curves. (**a**) *g*_*exc*_ = 0.0 *μ*S/cm^2^; (**b**) *g*_*exc*_ = 0.5 *μ*S/cm^2^; (**c**) *g*_*exc*_ = 2.0 *μ*S/cm^2^. SubH represents subcritical Hopf bifurcation. BHom represents saddle homoclinic orbit bifurcation. ${{\rm{SN}}}_{{\rm{2}}}^{{\rm{S}}}$ represents saddle node bifurcation of equilibrium.

The I–V curve at steady-state exhibited ∂*I*/∂*V* < 0 at SubH, ∂*I*/∂*V* > 0 at BHom, and ∂*I*/∂*V* = 0 at ${{\rm{SN}}}_{{\rm{2}}}^{{\rm{S}}}$, as show in Fig. 7. These are consistent with the proof shown in the Methods section. Results showed that the transition of bifurcations corresponding to excitability/spiking could be induced by the competition between inward and outward currents, which was achieved by changes in the inward current mediated by the excitatory autapse.

#### Bifurcations and behaviors related to transitions from class II to I spiking in the two-dimensional parameter space

**Figure 8:**
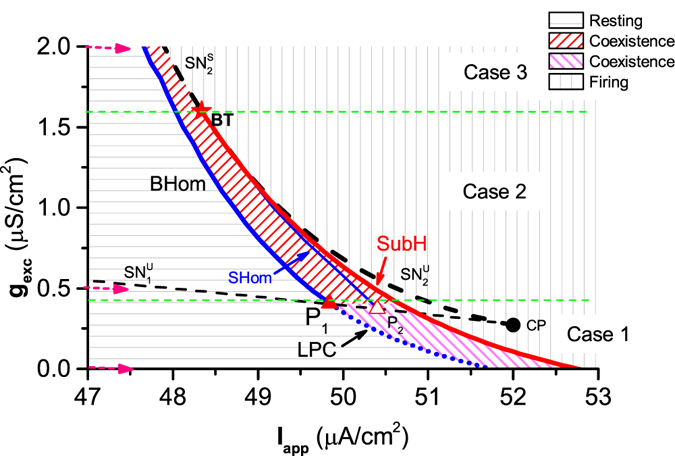
Two parameter bifurcations in the plane (*I*_*app*_, *g*_*exc*_) of the ML model with an excitatory autapse. SubH is subcritical Hopf curve. BHom and SHom are saddle homoclinic orbit bifurcation curves. ${{\rm{SN}}}_{{\rm{1}}}^{{\rm{S}}}$, ${{\rm{SN}}}_{{\rm{1}}}^{{\rm{U}}}$, ${{\rm{SN}}}_{{\rm{2}}}^{{\rm{S}}}$ and ${{\rm{SN}}}_{{\rm{2}}}^{{\rm{U}}}$ are curves of saddle node bifurcation of equilibrium. LPC represents the curve of saddle node bifurcation of limit cycle. CP is cusp bifurcation. BT is Bogdanov-Takens bifurcation.

We acquired the dynamic behaviors, codimension-1 bifurcation curves, and special and codimension-2 bifurcation points in the 2-dimensional parameter space (*I*_*app*_, *g*_*exc*_) of the ML model with excitatory autapse (Fig. 8). The left border of the coexisting behavior contains the BHom and LPC curves, and the right border is composed of the SubH and ${{\rm{SN}}}_{{\rm{2}}}^{{\rm{S}}}$ Curves. The LPC curve changed to a BHom curve at the special point *P*_1_ (*I*_*app*_ ≈ 49.84 *μ*A/cm^2^, *g*_*exc*_ ≈ 0.41 *μ*S/cm^2^, red solid triangle), which induced the transition from class II spiking to class I spiking. Meanwhile, the bifurcation curve SubH changed to ${{\rm{SN}}}_{{\rm{2}}}^{{\rm{S}}}$ at the codimension-2 bifurcation point BT (*I*_*app*_ ≈ 48.34 *μ*A/cm^2^, *g*_*exc*_ ≈ 1.5977 *μ*S/cm^2^, red star); however, the excitability remained unchanged as class II. The horizontal green lines across *P*_1_ and BT are the border between case 1 and 2, and between case 2 and 3, respectively. The positions of the 3 examples of bifurcations shown in Fig. 6 correspond to the pink dotted arrows.

The bifurcation curves related to excitability/spiking classes induced by the excitatory autapse changed from upper-left to bottom-right, which was different from that induced by the inhibitory autapse (Fig. 5). In addition, the bifurcation points *P*_2_ (the red open triangle) and CP (the red open circle), and the codimension-1 bifurcation curves ${{\rm{SN}}}_{{\rm{1}}}^{{\rm{U}}}$, ${{\rm{SN}}}_{{\rm{2}}}^{{\rm{U}}}$ (the black dashed line), and SHom, had no direct relationship to the transition between classes of excitability and spiking, and thus was not introduced in the present paper.

## Discussion

Two classes of neuronal excitability/spiking exhibit different electrophysiological properties and nonlinear dynamics that may have different effects in both single neurons and networks^2, 7–9^. This has been one of the most important subjects in both neuroscience and neurodynamics^1–3^. For example, the transition between classes of neuronal excitability/spiking have been observed in biological experiments, and can be used to interpret physiological phenomenon such as the resonance of pyramidal neurons at theta frequency, and symptoms of demyelination^16, 62^. The neuronal excitability/spiking excitability is classified into different classes by distinct firing frequency responses to external stimulation, which is determined by bifurcations^1–3, 16, 20–24, 28, 62^ in nonlinear dynamics and the balance between the inward and outward currents across the membrane^16, 19^. Class I excitability is related to a nearly 0 firing frequency, while class II excitability is related to a non-zero firing frequency. Transition between these 2 classes, which is induced by the modulation of ionic currents^2, 16, 19–28^, have been investigated with bifurcations and steady-state I–V curves^2, 19^.

It is well known that class I excitability is related to SNIC bifurcation, wherein the nullcline $\dot{V}=0$ is tangent to the nullcline $\dot{w}=0$, and ∂*I*/∂*V* = 0. Conversely, class II excitability is associated with SubH bifurcation, wherein the nullcline $\dot{V}=0$ intersects with the nullcline $\dot{w}=0$, and ∂*I*/∂*V* < 0. The transition between classes of neuronal excitability is related to codimension-2 bifurcation BT^2, 16, 19–28^. In the present paper, we found a comprehensive relationship between classes of neuronal excitability/spiking and the codimension-1 bifurcations, codimension-2 bifurcations, nullclines, and steady-state I–V curves. Firstly, class I excitability had a relationship to codimension-1 bifurcations such as SNIC, while class II excitability was associated with codimension-1 bifurcations such as SN, SubH, and SupH. Secondly, the nullcline $\dot{w}=0$ was tangent to the nullcline $\dot{V}=0$ for SNIC and SN, and intersected with the nullcline $\dot{V}=0$ for SubH, SupH, and BHom. Thirdly, class I spiking was related to codimension-1 bifurcations such as SNIC and BHom, while class II spiking was related to codimension-1 bifurcations such LPC and SupH. Fourthly, we identified the relationships between steady-state I–V curves and 5 types of codimension-1 bifurcations. For SNIC and SN, ∂*I*/∂*V* = 0^3, 19^. For SubH and SupH, ∂*I*/∂*V* < 0^3, 19^. For supercritical saddle Homoclinic bifucation BHom, ∂*I*/∂*V* > 0. Lastly, we found that multiple codimension-2 bifurcation points, including the BT, CP, and GH points, or special points *P*_1_ and *P*_3_, were related to classes of excitability or spiking.

In the present paper, we found that the inhibitory and excitatory autapses regulate the classes of neuronal excitability/spiking. Different from previous studies wherein the modulations to a neuron are external, the inhibitory autapse and excitatory autapse of a neuron provided “negative” (outward) current and “positive” (inward) current to the neuron via self-feedback, which plays different roles on the generation of an action potential^2, 16, 19, 31^. Autaptic currents have the same roles as some ion currents with same direction across the membrane. For example, the inhibitory autapse induced the transition from class I excitability/spiking to class II excitability/spiking, which is like other outward currents such as M-type potassium currents; meanwhile, the excitatory autapse induced the transition from class II spiking to class I spiking, resembles other inward currents, such as L-type calcium currents^2, 16, 19, 31^. The autaptic current has the same effect on variation of net current as those of blocking ion currents in experiments. For example, inhibitory autapse induces the change from class I to class II, which is consistent with blocking low threshold calcium currents and persistent sodium currents in spinal lamina I neurons^19^; excitatory autapse induces the change from class II to class I, which is consistent with blocking potassium currents in mesencephalic neurons, spinal lamina I neurons and cortical interneuron^19, 63^. In addition, experiments show that pyramidal and dopamine neurons *in vivo* exhibit activities different from those *in vitro*, which may be due to synaptic input which changes the net current^16, 27^.

The classes of neuronal excitability are influenced by the direction and magnitude of the net current active at perithreshold potentials^4, 16, 19^. The net current at perithreshold potentials for class I, II and III are weak inward, weak outward and strong outward currents^4, 16, 19^, respectively. The switches of neuronal excitability caused by regulating ion currents have been studied extensively. Experiment shows that the excitability of mesencephalic neurons switches from class III to class II and to class I through blocking potassium currents (outward current)^63^. Therefore, it can be speculated that autapse may also induce transition between classes of neuronal excitability related to class III excitability. For example, when an inhibitory autapse is introduced to ML model with class I excitability, the net current at perithreshold potentials may change from weak inward current (class I) to weak outward current (class II) and to strong outward current (class III) as autaptic strength increases. When an excitatory autapse is introduced to ML model with class III excitability, the net current at perithreshold potentials may change from strong outward current (class III) to weak outward current (class II) and to weak inward current (class I) as autaptic strength increases.

Based on the progress of the present paper, there are several questions to be answered in the future studies. Firstly, noise is ubiquitous in nervous systems and can induce phenomenon different from the deterministic system^64–69^. For example, stochastic integer multiple firing patterns and on-off firing patterns have been identified induced by noise near super-critical and sub-critical Hopf bifurcations, respectively, and have been related to coherence resonance^66–69^. Correspondingly, the frequency curve also changes. It can be speculated that complex stochastic dynamics appear near the bifurcation points when noise is introduced, which awaits further studied in future. Secondly, time delay is another important factor for the autapse, which can influence the activities of neurons such as firing patterns and firing region^32, 45–59^. When time delay is introduced, more complex bifurcations will be induced and should be calculated in future investigated. Lastly, the paper present dynamic mechanisms and biophysical basis of the autapses that can regulate the classes of the neuronal excitability/spiking, which awaits the experimental demonstration in future investigation.

## Materials and Methods

### The Morris-Lecar (ML) model and the autapse model

The ML model is widely used to investigate class I excitability and class II excitability, and is described as follows^3, 10, 20^:

1 $$C\dot{V}(t)=-{g}_{Ca}{m}_{\infty }(V(t))\,(V(t)-{V}_{Ca})-{g}_{k}w(t)\,(V(t)-{V}_{K})-{g}_{L}(V(t)-{V}_{L})+{I}_{app},$$

2 $$\dot{w}(t)=\varphi \frac{{w}_{\infty }(V(t))-w(t)}{{\tau }_{w}(V(t))}.$$

where *C* is the membrane capacitance, and *V*(*t*) and *w*(*t*) are the membrane voltage and activation of delayed rectified *K* ^+^ current, respectively. The first 3 terms in the right-hand side of Eq. 1 represent the *Ca* ^2+^ current, the voltage-gated delayed-rectifier *K* ^+^ current, and the leak current. Parameters *g*_*Ca*_, *g*_*K*_ and *g*_*L*_ are the maximal conductances of the calcium current, potassium current, and leak current, respectively. *V*_*Ca*_ and *V*_*K*_ and *V*_*L*_ are the reversal potentials of the calcium current, potassium current, and leak current, respectively. Lastly, *I*_*app*_ is the applied (depolarization) current. ${m}_{\infty }(V)=0.5\,[1+\,\tanh \,((V-{V}_{1})/{V}_{2})]$, ${\tau }_{w}(V)={[\cosh ((V-{V}_{3}\mathrm{)/2}{V}_{4})]}^{-1}$, ${w}_{\infty }(V)=0.5\,[1+\,\tanh \,((V-{V}_{3})/{V}_{4})]$.

The parameters are set as follows: *C* = 20 *μ*F/cm^2^, *V*_*K*_ = −84 mV, *V*_*Ca*_ = 120 mV, *V*_*L*_ = −60 mV, *g*_*K*_ = 8 *μ*S/cm^2^, *g*_*Ca*_ = 4 *μ*S/cm^2^, *g*_*L*_ = 2 *μ*S/cm^2^, *V*_1_ = −1.2 mV, *V*_2_ = 18 mV, *V*_4_ = 17.4 mV, *ϕ* = 0.067. Furthermore, *V*_3_ = 12 mV for class I excitability, and *V*_3_ = 2 mV for class II excitability.

The autapse model, which was based the sigmoid function of the presynaptic voltage with a threshold Θ_*syn*_, is described by the following equation^70^:

3 $${I}_{aut}={g}_{s}({V}_{pos}-{V}_{syn})\,{\rm{\Gamma }}({V}_{pre}-{{\rm{\Theta }}}_{syn}).$$

where ${\rm{\Gamma }}({V}_{pre}-{{\rm{\Theta }}}_{syn})=\mathrm{1/}(1+exp(-({V}_{pre}-{{\rm{\Theta }}}_{syn})/\lambda ))$ is the sigmoidal function, *V*_*pre*_ and *V*_*pos*_ are presynaptic and postsynaptic membrane potentials, respectively, Θ_*syn*_ is the synapse threshold, and *V*_*syn*_ is the reversal potential. Θ_*syn*_ was set −20 mV to ensure that a spike can cross it. *V*_*syn*_ was set 10 mV for excitatory autapses, and −60 mV for inhibitory autapses. *g*_*s*_ is the synaptic strength, where the subscript “s” is “exc” for the excitatory autapse and “inh” for the inhibitory autapse. *V*_*pre*_ = *V*_*pos*_ = *V* to ensure that the synapse is autapse. Finally, *λ* determines the release rate of neurotransmitters, and was set as 1 for the inhibitory autapse, and 2 for the excitatory autapse.

## Methods

The ML model was integrated with the Runge-Kutta method with a time step of 0.001 ms. Bifurcations were acquired using the bifurcation toolbox Matcont^71^ and XPPAUT^72^.

For the ML model, the steady state I–V curve is described as

$$\begin{array}{rcl}I(V) & = & -{I}_{Ca}-{I}_{K}-{I}_{L}-{I}_{aut}\\  & = & -{g}_{Ca}{m}_{\infty }(V-{V}_{Ca})-{g}_{K}w(V-{V}_{K})-{g}_{L}(V-{V}_{L})-{g}_{aut}(V-{V}_{syn})\,{\rm{\Gamma }}(V)\\  & = & -{g}_{Ca}{m}_{\infty }(V-{V}_{Ca})-{g}_{K}{w}_{\infty }(V-{V}_{K})-{g}_{L}(V-{V}_{L})-{g}_{aut}(V-{V}_{syn})\,{\rm{\Gamma }}(V).\end{array}$$

and then

4 $$\frac{\partial I}{\partial V}=-\frac{\partial {I}_{Ca}}{\partial V}-\frac{\partial {I}_{K}}{\partial V}-\frac{\partial {I}_{K}}{\partial w}\frac{\partial {w}_{\infty }}{\partial V}-\frac{\partial {I}_{L}}{\partial V}-\frac{\partial {I}_{aut}}{\partial V}.$$

The characteristics of the steady-state I–V curve corresponding to different bifurcations were acquired by stability analysis of steady-state and linearization to the ML model.

The ML model with autapse can be described as follows:

5 $$\dot{V}(t)={C}^{-1}({I}_{app}-{I}_{Ca}(t)-{I}_{K}(t)-{I}_{L}(t)-{I}_{aut}(t))={C}^{-1}({I}_{app}+I(V)),$$

6 $$\dot{w}(t)=\varphi \frac{{w}_{\infty }(V(t))-w(t)}{{\tau }_{w}(V(t))}=g(V,w).$$

Suppose that (V_*_, w_*_) is the equilibrium of the ML model with autapse, then, the Jacobian matrix *J* of the model at resting state (V_*_, w_*_) is as follows:

$$J=(\begin{array}{cc}{C}^{-1}\frac{\partial (-{I}_{Ca}-{I}_{K}-{I}_{L}-{I}_{aut})}{\partial V} & \frac{\partial g}{\partial V}\\ {C}^{-1}\frac{\partial {I}_{K}}{\partial w} & \frac{\partial g}{\partial w}\end{array}),$$

where $\frac{\partial g}{\partial V}=\frac{\varphi }{{\tau }_{w}}\frac{\partial {w}_{\infty }}{V}$ and $\frac{\partial g}{\partial w}=-\frac{\varphi }{{\tau }_{w}} < 0$. Then the character equation is described as:

$$\begin{array}{rcl}Det(J) & = & {\lambda }^{2}+({C}^{-1}\frac{\partial ({I}_{Ca}+{I}_{K}+{I}_{L}+{I}_{aut})}{\partial V}-\frac{\partial g}{\partial w})\lambda \\  &  & -\frac{\partial g}{\partial w}({C}^{-1}\frac{\partial ({I}_{Ca}+{I}_{K}+{I}_{L}+{I}_{aut})}{\partial V})+{C}^{-1}\frac{\partial {I}_{K}}{\partial w}\frac{\partial g}{\partial V}\\  & = & {\lambda }^{2}+b\lambda +c\mathrm{.}\end{array}$$

where $b=({C}^{-1}\frac{\partial ({I}_{Ca}+{I}_{K}+{I}_{L}+{I}_{aut})}{\partial V}-\frac{\partial g}{\partial w})$ and $c=-\frac{\partial g}{\partial w}\,({C}^{-1}\frac{\partial ({I}_{Ca}+{I}_{K}+{I}_{L}+{I}_{aut})}{\partial V})+{C}^{-1}\frac{\partial {I}_{K}}{\partial w}\frac{\partial g}{\partial V}$. Then, we obtained the characteristics of the steady-state I–V curve based on the necessary condition for the different bifurcations of equilibrium.

1. If the equilibrium (V_*_, w_*_) is saddle-node or SNIC bifurcation, then *b* and *c* satisfy *b* > 0 and *c* = 0. According to Eq. 4, and thus, $c={C}^{-1}\frac{\varphi }{{\tau }_{w}}(-\frac{\partial I}{\partial V})$, and then ∂*I*/∂*V* = 0. Therefore, ∂*I*/∂*V* = 0 is the necessary condition for SNIC bifurcation or saddle-node bifurcation.
2. If the equilibrium (V_*_, w_*_) is Hopf bifurcation, then *b* and *c* satisfy *b* = 0 and *c* > 0. From *c* > 0, we can acquire ∂*I*/∂*V* < 0. This indicates that ∂*I*/∂*V* < 0 at the equilibrium (V_*_, w_*_) is the necessary condition for Hopf bifurcation.
3. If the equilibrium (V_*_, w_*_) is saddle homoclinic bifurcation, then *b* and *c* satisfy *b* < 0 and *b* ^2^ − 4*c* > 0 at the saddle, and the saddle quantity equals *c*. If the saddle homoclinic bifurcation is supercritical^2, 73^, the saddle quantity is negative, i.e. *c* < 0 for the BHom, which implies that ∂*I*/∂*V* > 0 at equilibrium (V_*_, w_*_) is the necessary condition for supercritical saddle homoclinic bifurcation.
